# Susceptibility of *Malassezia pachydermatis* Clinical Isolates to Allopathic Antifungals and Brazilian Red, Green, and Brown Propolis Extracts

**DOI:** 10.3389/fvets.2019.00460

**Published:** 2019-12-13

**Authors:** Kathleen Ramos Deegan, Maisa Santos Fonseca, Diogo Coelho Pádua Oliveira, Laerte Marlon Santos, Clara Couto Fernandez, Samira Abdallah Hanna, Bruna Aparecida Souza Machado, Marcelo Andrés Umsza-Guez, Roberto Meyer, Ricardo Wagner Portela

**Affiliations:** ^1^Instituto de Ciências da Saúde, Universidade Federal da Bahia, Salvador, Brazil; ^2^Instituto de Ciências Biológicas, Universidade Federal de Minas Gerais, Belo Horizonte, Brazil; ^3^Instituto de Tecnologias da Saúde, Centro Universitário SENAI-CIMATEC, Salvador, Brazil

**Keywords:** antifungals, azoles resistance, canine malasseziosis, ethanolic extraction, supercritical extraction

## Abstract

Clinical mycoses treatment is associated with issues such as negative side effects, high cost, prolonged treatment, and resistant strain selection. *Malassezia pachydermatis* is the most frequently isolated yeast in cases of canine otitis and dermatitis. The number of fungal strains exhibiting primary resistance to several drugs *in vitro* is increasing. Propolis has a diverse chemical composition and well-known therapeutic properties against mycoses. An alternative method for producing propolis extracts using supercritical fluid has higher selectivity, yielding extracts with fewer pollutant residues. This study therefore aimed to evaluate the *in vitro* susceptibility profile of *M. pachydermatis* clinical isolates to precharacterized supercritical and ethanolic extracts. Three types of Brazilian propolis extracts (green, red, and brown) and commercial allopathic antifungals were used in this investigation. We used the microdilution broth technique to evaluate the susceptibility profile of the yeasts. The minimum inhibitory concentration (MIC) of the brown propolis ethanolic extract was ≥16 μg/mL for all isolates. The MICs of fluconazole, ketoconazole, itraconazole, and amphotericin B ranged from 8 to >64 μg/mL, 0.032–4 μg/mL, 0.0313–16 μg/mL, and 1–2 μg/mL, respectively. The MICs of ethanolic red propolis extracts were lower than those of supercritical red propolis extracts. However, the green propolis ethanolic extract had more pronounced fungicidal activity. Isolates with lower susceptibility to commercial fungicides were inhibited by red and green propolis extracts. These results indicate that propolis can potentially be used in *in vivo* experiments as a promising therapeutic agent against *M. pachydermatis* infections.

## Introduction

Propolis is a complex, resinous, and balsamic product produced by bees during the collection of resins from shoots, exudates, and other plant tissues. It contains additives such as salivary secretions, wax, and pollen (1). Propolis is used as a building material and defensive substance; additionally, it is used to embalm corpses in hives. It ensures lower contamination by microorganisms from the external environment (2). Brazilian propolis are classified into 13 types based on their geographical origin and physicochemical properties, such as color, texture, and chemical profile (3–5). Its chemical composition and biological activity depends on the climate, flora, bee species, environmental conditions, and collection period (3, 6–8).

The main compounds isolated from propolis include aliphatic acids and esters, aldehydes and aromatic esters, sugars, alcohols, fatty acids, amino acids, steroids, ketones, chalcones, flavonoids, terpenes, lignans, polyphenols, proteins, vitamins, and minerals (3, 4, 8–16). The biological and pharmacological activities of propolis and its ability to function as an antifungal (17–21), antimicrobial (12, 15, 22, 23), healing (24), analgesic (25), immunomodulatory (26), antiviral (27), anti-inflammatory (14), hepatoprotective (28), antiulcerogenic (29), antiparasitic (30, 31), anticarcinogenic (11, 12, 30), and antioxidant (9, 10, 13, 30, 31) agent depends on its unique chemical composition. The most common technique for obtaining propolis extract is ethanolic extraction via maceration. However, alternative solvents have been used for extraction (15, 32, 33). Alternative techniques such as ultrasound pretreatment and supercritical fluid extraction are more efficient for effectively obtaining phenolic compounds, flavonoids, antioxidants, and cinnamic acid derivatives (34, 35).

*Malassezia pachydermatis* is the most commonly isolated fungi in dogs with external otitis or dermatitis (36, 37). In animals, *Malassezia* infections are often treated using topical or systemic azole derivatives (38). In most cases, these infections are recurrent (36). The emergence of *M. pachydermatis* isolates that weakly respond to azoles (39–44) emphasizes the importance of susceptibility testing for deciding appropriate treatment courses.

As this yeast is considered an important etiological agent of dermatomycoses in veterinary clinics, this adds to the disadvantages associated with allopathic antifungal therapy. Owing to increasing azole resistance, few studies have explored the therapeutic potential of Brazilian propolis extracts against this lipophilic yeast (45, 46). The current study therefore aimed to verify the susceptibility of clinical isolates of *M. pachydermatis* to allopathic antifungals and ethanolic and supercritical Brazilian propolis extracts.

## Materials and Methods

### Origin, Processing, and Characterization of Propolis Samples

The origin, processing, and characterization of Brazilian propolis samples have been described previously (10). Red (RAL), brown (BSC), and green (GPR) propolis were obtained from the Alagoas, Santa Catarina, and Paraná states of Brazil.

The propolis samples were ground in a grinder and sieved (60 mesh) to obtain particles of suitable size (~0.250 mm). This was done to increase the surface area of the samples. Samples were homogenized to initiate the extraction process. The protein content, ash content, total lipid content, mineral content, and the humidity and water activity of the samples were already determined (10). These values are shown in Supplementary Material 1.

### Propolis Extraction Methodologies

The process for extract identification, the conditions used during each extraction process, and the chemical composition of red, green, and brown propolis extracts (10) are shown in Supplementary Material 2.

#### Conventional Ethanolic Extraction

First, 15 mL ethanol (80%) was added to 2 g of propolis. Extraction was performed at 70°C for 30 min while stirring the solution constantly in a shaker incubator at 710 rpm. The extract was centrifuged at 5,000 *x* g for 11 min at 4°C. The supernatant was then transferred to a 50 mL vessel. Following this, 10 mL ethanol (80%) was added to the residue in the tube and centrifugation was repeated. Supernatants were homogenized and dried thoroughly at 50°C. Extracts were stored in tubes covered with aluminum foil under inert atmospheric conditions (N_2_) to avoid material degradation.

#### Supercritical Fluid Extraction (CO_2_)

An SFT-110 Supercritical Fluid Extractor (Supercritical Fluid Technologies, Inc.) pilot unit was used to obtain the extracts. The equipment was composed of a high-pressure bomb (capacity up to 10.000 psi), extraction cell (capacity 100 mL), oven (with a preheater), static/dynamics and restrictor valves, flow meter, and a CO_2_ cylinder. A CO_2_ cylinder with a fishing tube was used to ensure that CO_2_ was used only in a liquid state in the system. The extraction cell was then assembled and maintained at 40°C. The supercritical extraction process was performed at 40°C, 300 bar, a 110 mass of CO_2_/mass of propolis ratio, and 1% co-solvent (ethanol m/m). The CO_2_ output in the system was 6.0 g/min in all experiments, and the total time required for extraction was ~2.5 h (35).

### Microorganisms

Twelve *M. pachydermatis* isolates from dogs with otitis (03) or fungal dermatitis (06) and from a wild *Didelphis aurita* with otitis (03) were included in this study. These isolates were included in the Fungi Collection of the Laboratory of Mycosis at the Veterinary Hospital of the Federal University of Bahia, which donated them for this study. The BH3 strain, which had been previously characterized (47) was used as a reference strain. This strain was kindly provided by Prof. Patrícia Cisalpino (Institute of Biological Sciences, Federal University of Minas Gerais). The *M. pachydermatis* isolates were cultivated in petri dishes containing Sabouraud dextrose agar supplemented with 0.5% extra virgin olive oil and 0.5% Tween 80. The plates were incubated at 34–37°C for 72 h (48).

The *M. pachydermatis* isolates were characterized phenotypically and physiologically as described by Guillot et al. (49). To confirm the identifications, the ITS and nuclear large subunit rDNA (LSU) regions were sequenced. Briefly, the genomic DNA of the clinical isolates was extracted using the FastDNA Spin Kit (Mp Biomedicals, Solon, OH, USA). Polymerase chain reaction (PCR) was performed using ITS4 and ITS5 primers for the amplification of the complete internal transcribed region 26, and LROR and LR7 primers for the amplification of the LSU. All PCR reactions were performed using Quatro G Taq DNA polymerase (Porto Alegre, RS, Brazil) in a final volume of 50 μL, containing 10 μL Quatro G buffer, 3.0 μL MgCl 2 (50 mM), 1 μL DNTP (10 mM), 1 μL forward primer (10 pmol), 1 μL reverse primer (10 pmol), 1 μL DMSO, 1.5 μL BSA (1 μg/μL), 5 μL Betaine (5 M), 0.2 μL Taq 5 U/μL), 24.8 μL sterile water, and 1 μL DNA template. The reactions were carried out in a thermocycler as per the following process: 2 min at 94°C, 35 cycles of 1 min at 94°C, 1 min at 55°C, 1 min at 72°C, and a final 5 min extension at 72°C. The PCR product purification was carried out using an ethanol/EDTA 125 mM precipitation protocol. The DNA sequencing was executed on ABI 3730 automated sequencer (Applied Biosystems, Life Technologies, Carlsbad, CA, USA). Consensus sequences were submitted to the Basic Local Alignment Search Tool (BLAST) for identification by similarity analysis with the nucleotide sequences database of GenBank of the National Center for Biotechnology Information (NCBI).

### Antifungal Activity: Determining the Minimal Inhibitory Concentration and Minimal Fungicidal Concentration

Sabouraud dextrose broth supplemented with 1% Tween 80 was selected for performing the tests. In recent studies on the standardization of microbial techniques for the genus *Malassezia*, this has been described as the most suitable medium (50). The following antifungal drugs were used in this study: itraconazole (22% pellets, Infinity Pharma, Campinas, SP, Brazil), fluconazole (powder ≥ 98%, Infinity Pharma, Campinas, SP, Brazil), ketoconazole (powder ≥ 98%, Infinity Pharma, Campinas, SP, Brazil), and amphotericin B (injectable ampoule powder 50 mg, Cristália, São Paulo, SP, Brazil). The antifungal solutions were prepared as recommended by the reference CLSI broth microdilution M27-A3 protocol (51), using DMSO (Êxodo Científica, Sumaré, SP, Brazil) as a diluent. To evaluate the minimal inhibitory concentration (MIC) and minimal fungicidal concentration (MFC), the following antifungal agents were used: 0.125–64 μg/mL fluconazole and 0.0313–16 μg/mL ketoconazole, itraconazole, and amphotericin B (51). The propolis extract concentrations ranged from 0.313–16.0 mg/mL. The microbiological growth of the extracts was controlled by cultivation in Müller-Hinton broth, using sterile 96-well microplates (Thermo Fisher Scientific, Waltham, MA), as previously recommended (51).

The inocula were diluted in sterile saline solutions. Using a spectrophotometer, its absorbances at 530 nm were found to be 0.180 and 0.220 nm. These values were used to determine the cell concentration. This procedure resulted in suspensions with a concentration of ~3.0 × 10^8^ CFU/mL. The inoculum was then diluted 1:20 in culture medium. One hundred microliters of each antifungal was diluted in the culture medium twice, and 100 μL of the inoculum was added into each well. The final concentration of the inoculum was 7.5 × 10^6^ CFU/mL. All tests were performed in duplicate. The controls, which indicated the sterility and viability of the inoculum, were also assayed as previously recommended (51). Readings were obtained at 625 nm using a plate reader (Thermo Scientific, Waltham, MS) after performing incubation for 72 h at 35°C. To determine the MFC, aliquots of 10% (20 μL) of the total well-volume of each tested antifungal were seeded in petri dishes containing Sabouraud dextrose agar. After incubating wells for 72 h at 35°C, the presence or absence of fungal growth was noted to determine the MFC. Negative controls were comprised of the medium without any added inoculum, the diluent, and each of the propolis extracts with different concentrations. Positive controls were assayed using media that did not contain the antifungal agent and inoculum.

### Data Analysis

In the broth microdilution tests, the growth percentage of each extract was calculated as an increase in optical density/turbidity relative to that of the negative control of each extract concentration. The optical density of the positive control of each isolate present, along with the culture medium and inoculum alone, was considered the maximum value. This was used to calculate the growth of each isolate in the test wells. In summary, percentage inhibition was calculated using the following formula:

$$\begin{array}{l} \text{Inhibition}\left(\%\right)=1-\left(\frac{\frac{\text{TWx}-\text{WNC}}{\text{WNC}}}{\frac{\text{PCx}-\text{NC}}{\text{NC}}}\right) \end{array}$$

where:

TWx = Optical density of the well-containing each antifungal solution with a different concentration (culture medium, fungal inoculum, and antifungal).
WNC = Optical density of the negative control, represented by each antifungal solution with a different concentration, to which fungal inoculum was not added.
PCx = Optical density of the positive control well (culture medium + fungal inoculum), to which no antifungal was added.
NC = Optical density of the negative control well (culture medium only).

The MIC was considered to be the drug concentration capable of inhibiting 90% of yeast growth. The MFC was considered to be the minimum drug concentration that resulted in complete killing of the yeast inoculum. The cut-off values for isolate classification, in regards to susceptibility or resistance to each antifungal, were as follows: 0.5 μg/mL for ketoconazole (52); 0.25 μg/mL for itraconazole (52); 1.0 μg/mL for amphotericin B (53); and 32 μg/mL for fluconazole (50).

The graphs representing the inhibition curves for each treatment were designed using GraphPad Prism 6.01 software (San Diego, CA, USA). The inhibition percentages for each drug and extract concentration reflect the mean of two independent experiments.

The MFC/MIC ratio was calculated to determine whether the extracts exhibited fungicidal or fungistatic activity. When the MFC/MIC ratio was < 4, the drug was considered to have fungicidal effects. If the ratio was ≥ 4, it was considered a fungistatic agent (17).

The analysis of dose-response curves (drc) package, available in the “R” software, was used to estimate the inhibition curve for each treatment. This statistical regression model describes a parametric function representing the average of the observed responses (54). The log-logistic regression model for four parameters was chosen, because it was constructed based on data obtained from < 20 isolates. The concentration of each antifungal agent inhibiting 50% of the isolate growth (EC_50_) was estimated by data sets fitting this regression model.

## Results

Considering the cut-off values adopted herein, 46.15, 38.46, 30.77, and 53.85% of the isolates were classified as being resistant to fluconazole, ketoconazole, itraconazole, and amphotericin B, respectively (Table 1). We observed that 4 of the 13 isolates (30.77%) developed resistance to multiple azoles used herein. Two of these were isolated from the skin of dogs with clinical symptoms, and two from the ears. Two of these isolates were also resistant to amphotericin B (7.89%). Growth was inhibited by each concentration of the four antifungals, tested using the broth microdilution technique (Supplementary Materials 3–6). Amphotericin B was the antifungal that exhibited the greatest uniformity in inhibition among the isolates.

**Table 1:** The MIC and MFC (μg/mL) values and MFC/CIM ratio of commercial antifungals used against *Malassezia pachydermatis* isolates, obtained via broth microdilution.

**Isolate**	**Fluconazole**				**Ketoconazole**				**Itraconazole**				**Amphotericin B**			
	**MIC**	**MFC**	**MFC/** **MIC**	**Response**	**MIC**	**MFC**	**MFC/** **MIC**	**Response**	**MIC**	**MFC**	**MFC/** **MIC**	**Response**	**MIC**	**MFC**	**MFC/** **MIC**	**Response**
BH3	16	16	1	S	0.125	0.125	1	S	0.125	0.125	1	S	2	4	2	**R**
302	8	16	2	S	0.0625	0.0625	1	S	0.0313	0.313	1	S	2	4	2	**R**
304	64	>64	–	**R**	0.0625	0.0625	1	S	0.25	O.25	1	S	2	4	2	**R**
336	>64	>64	–	**R**	1	1	1	**R**	0.5	0.5	1	**R**	1	4	4	S
389	>64	>64	–	**R**	4	4	1	**R**	4	8	2	**R**	1	2	2	S
311	64	>64	–	**R**	1	2	2	**R**	0.25	1	4	S	2	4	2	**R**
175	16	64	4	S	0.032	0.0625	2	S	0.0313	0.0313	1	S	1	4	4	S
487	>64	>64	–	**R**	2	4	2	**R**	16	>16	–	**R**	2	2	1	**R**
476	>64	>64	–	**R**	2	2	1	**R**	1	1	1	**R**	2	4	2	**R**
523	32	64	2	S	0.25	0.25	1	S	0.0313	0.0625	2	S	1	4	4	S
262	32	32	1	S	0.0625	0.125	2	S	0.0313	0.0313	1	S	2	4	2	**R**
240	32	>64	–	S	0.125	0.125	1	S	0.0625	0.0625	1	S	1	4	4	S
241	32	64	2	S	0.0625	0.125	2	S	0.0625	0.0625	1	S	1	4	4	S

*Each M. pachydermatis isolate was classified as resistant (R) or sensitive (S) according to pre-established cutoff values. The resistance occurrence is highlighted in bold*.

**Figure 1:**
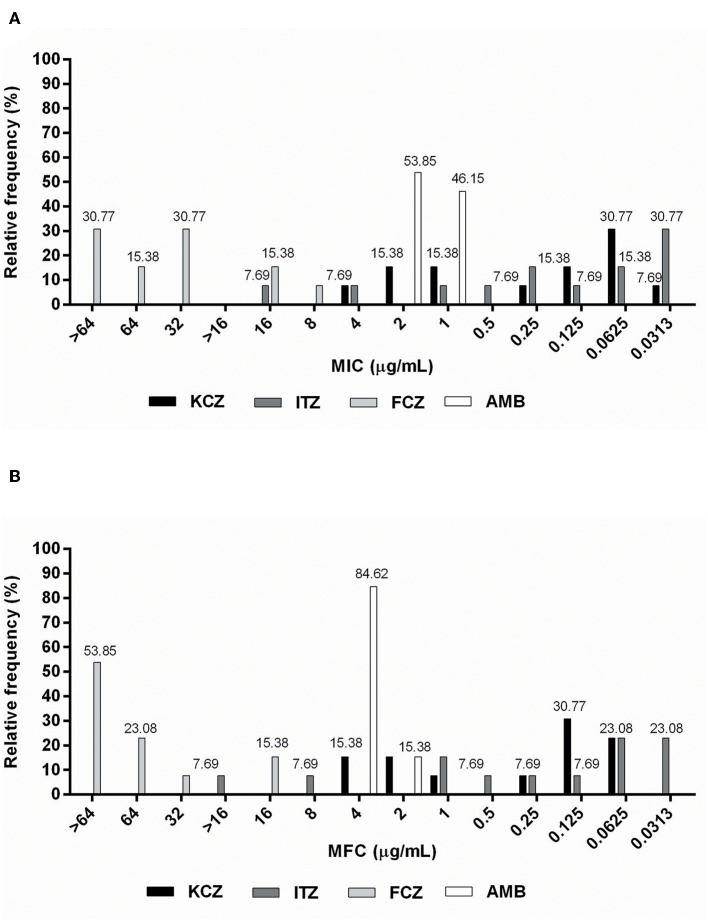
Relative frequency of **(A)** MIC (μg/mL) and **(B)** MFC (μg/mL) values obtained from *Malassezia pachydermatis* isolates against commercial allopathic antifungals.

The MIC values for fluconazole ranged from 8 μg/mL to ≥64 μg/mL, with MFC values ranging between 16 and ≥64 μg/mL (Table 1). In total, 46.15% of isolates developed resistance (Figure 1A) to fluconazole, which was considered the antifungal with the most number of isolates with undetermined MFCs (53.85%) (Figure 1B). MIC values for amphotericin B ranged from 1 to 2 μg/mL (Table 1), with 53.85% of the isolates having MIC values of 2 μg/mL (Figure 1A). These were classified as drug-resistant isolates. The MFC values of amphotericin B ranged from 2–4 μg/mL (Table 1), with 84.62% of the isolates having an MFC of 4 μg/mL (Figure 1B). The MIC values for itraconazole ranged from 0.313–16 μg/mL. It was found that its MFC values ranged from 0.313 μg/mL to > 16 μg/mL (Table 1), and had the lowest percentage (30.77%) of isolates classified as resistant (MIC > 0.25 μg/mL) (Figure 1A). The MIC values for ketoconazole ranged from 0.032–4 μg/mL, and its MFC ranged from 0.0625–4 μg/mL (Table 1). In total, 38.46% of isolates were resistant to ketoconazole (MIC > 0.5 μg/mL) (Figure 1A).

According to the parameter used to classify antifungal agents as fungicidal or fungistatic (17), the maximum fungicidal activity was shown by ketoconazole against 100% of the isolates. This was followed by itraconazole (76.92%), amphotericin B (61.54%), and fluconazole (38.46%). It was not possible to determine the relationship between the parameters in two isolates showing activity against itraconazole (15.38%), or in seven isolates tested with fluconazole (53.85%) (Table 1).

All propolis extracts inhibited the growth of *M. pachydermatis* isolates (Table 2). The growth inhibition curves for each type of propolis with different concentrations, tested using the broth microdilution technique, are shown in Supplementary Materials 7–10.

**Table 2:** The MIC and MFC (mg/mL) values and MFC/CIM ratio for each type of propolis extract used against *M. pachydermatis* isolates, determined via broth microdilution.

**Isolate**	**BSC–ET**			**GPR–ET**			**RAL–ET**			**RAL–SC**			**Antifungals**			
	**MIC**	**MFC**	**MFC/MIC**	**MIC**	**MFC**	**MFC/MIC**	**MIC**	**MFC**	**MFC/MIC**	**MIC**	**MFC**	**MFC/MIC**	**FLZ**	**KTZ**	**ITZ**	**AMB**
BH3	>16	>16	–	8	>16	–	8	>16	–	8	>16	–	S	S	S	**R**
302	>16	>16	–	8	16	2	4	8	2	8	16	2	S	S	S	**R**
304	16	>16	–	8	16	2	4	8	2	4	16	4	**R**	S	S	**R**
336	>16	>16	–	8	8	1	4	8	2	8	16	2	**R**	**R**	**R**	S
389	>16	>16	–	8	>16	–	4	8	2	8	8	1	**R**	**R**	**R**	S
311	>16	>16	–	8	16	2	4	8	2	4	16	4	**R**	**R**	S	**R**
175	>16	>16	–	8	16	2	4	8	2	8	8	1	S	S	S	S
487	>16	>16	–	4	4	1	4	16	4	4	16	4	**R**	**R**	**R**	**R**
476	>16	>16	–	8	16	2	8	16	2	8	8	1	**R**	**R**	**R**	**R**
523	16	>16	–	8	16	2	4	8	2	4	8	2	S	S	S	S
262	16	>16	–	4	8	2	4	8	2	4	16	4	S	S	S	**R**
240	16	>16	–	4	4	1	4	8	2	4	16	4	S	S	S	S
241	16	>16	–	8	16	2	4	8	2	4	16	4	S	S	S	S

*BSC–ET, brown propolis ethanolic extract from Santa Catarina, Brazil; GPR–ET, green propolis ethanolic extract from Paraná, Brazil; RAL–ET, red propolis ethanolic extract from Alagoas, Brazil; RAL–SC, red propolis supercritical extract from Alagoas, Brazil. FLZ, fluconazole; KTZ, ketoconazole; ITZ, itraconazole; AMB, amphotericin B; R, resistant; S, sensitive. The resistance occurrence is highlighted in bold*.

**Figure 2:**
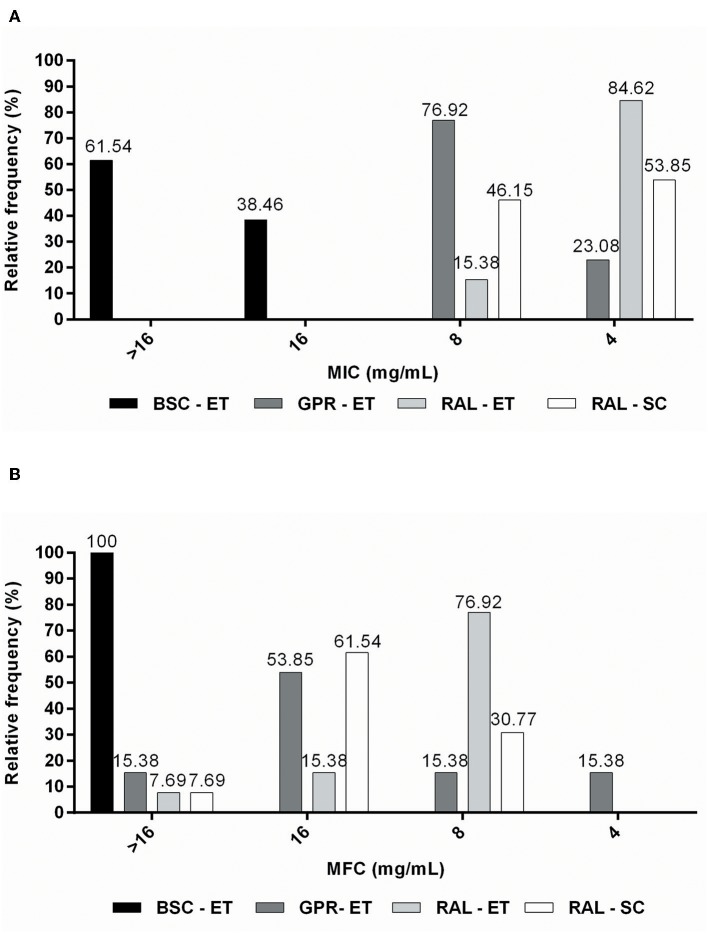
Relative frequency of **(A)** MIC (mg/mL) and **(B)** MFC (mg/mL) values obtained from *M. pachydermatis* isolates against propolis extracts.

The red propolis ethanolic extract exhibited activity against the highest proportion of isolates (84.62%), with the MIC being the lowest for propolis extracts (4 mg/mL) (Figure 2A). The supercritical red propolis extract showed the second highest inhibitory activity, with 53.85% of isolates exhibiting an MIC of 4 mg/mL (Figure 2A). With a lower proportion of isolates sensitive to a concentration of 4 mg/mL (23.08%), the green propolis ethanolic extract had a higher yeast concentration (76.92%) and had an MIC of 8 mg/mL (Figure 2A). Finally, the brown propolis ethanolic extract had the highest inhibitory concentrations (MIC > 16 mg/mL) in most tests (61.54%), and the worst inhibitory activity among the propolis extracts (Figure 2A).

The green propolis ethanolic extract was the only one that demonstrated fungicidal effects at a concentration of 4 mg/mL (15.38% of isolates) (Figure 2B). Red ethanolic propolis exhibited activity against the highest proportion (76.92%) of isolates, and had an MFC of 8 mg/mL (Figure 2B). The supercritical red propolis extract was able to kill 30.77% of isolates at a concentration of 8 mg/mL, and its highest fungicidal effects (61.54%) were observed at a concentration of 16 mg/mL (Figure 2B). Brown propolis ethanolic extracts with higher MFC values did not exhibit lethality at the highest tested concentrations (Figure 2B). MFC/MIC ratio analysis demonstrated that greater fungicidal effects were observed for the ethanolic green propolis extract, followed by the ethanolic and supercritical red propolis extracts (Table 2).

**Figure 3:**
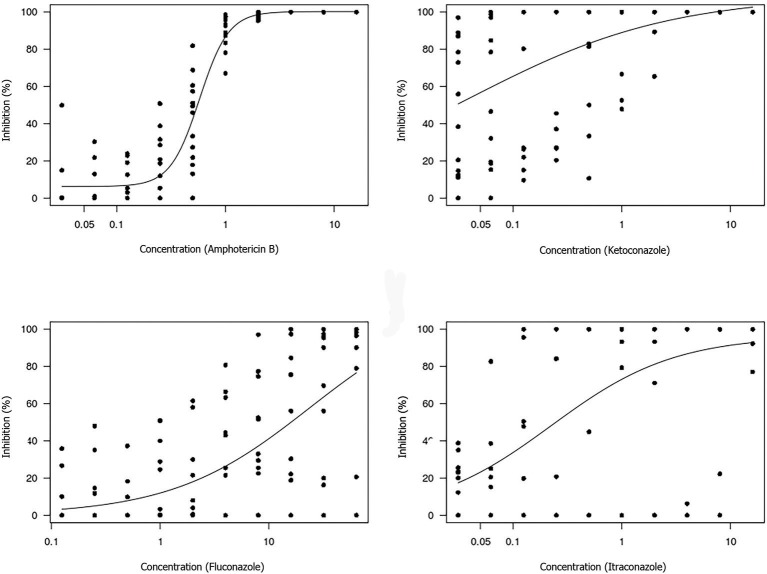
Dose-response curve for each antifungal tested using the broth microdilution technique to determine the EC50 value of *M. pachydermatis* isolates. The results are expressed in μg/mL.

**Figure 4:**
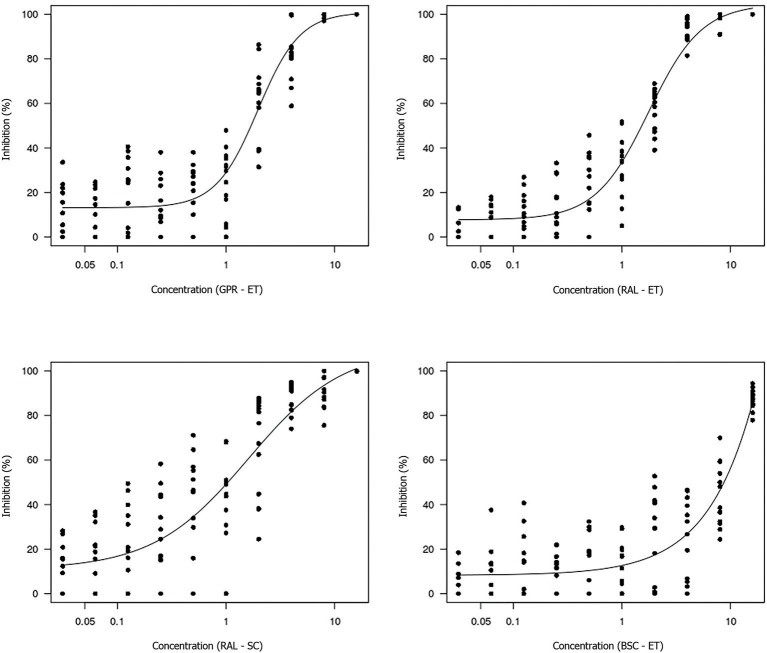
Dose-response curve for each propolis extract tested using the broth microdilution technique for determining the EC_50_ value of *M. pachydermatis* isolates. GPR–ET, green propolis ethanolic extract from Paraná, Brazil; RAL–ET, red propolis ethanolic extract from Alagoas, Brazil; RAL–SC, red propolis supercritical extract from Alagoas, Brazil; BSC–ET, brown propolis ethanolic extract from Santa Catarina, Brazil. The results are expressed in mg/mL.

The non-linear log-logistic regression model was used to construct a dose-response curve for each antifungal agent (Figures 3, 4). Amphotericin B was the only antifungal drug fitting this model. Among all the extracts, only the brown propolis ethanolic extract did not fit the proposed model. The EC_50_ value could not be determined for antifungal agents exhibiting a large dispersion of data and did not fit the model. The ethanolic extracts of green and red propolis presented a similar curve pattern, potentially indicating the similarity of their inhibition mechanisms. The decrease in the dose-response curve modeled for red propolis supercritical extract was lower, as shown by its lower EC_50_ values.

The estimated concentrations for 50% isolate growth inhibition (EC_50_) were 0.58 μg/mL amphotericin B, 1.97 mg/mL green propolis ethanolic extract, 1.76 mg/mL red propolis ethanolic extract, and 1.60 mg/mL red propolis supercritical extract. The red propolis supercritical extract inhibited 50% of isolate growth at a lower concentration, although it exhibited greater data dispersion when compared to the ethanolic extracts of red and green propolis. The small curve decay indicated a lower dose-dependent response.

## Discussion

The low response of *M. pachydermatis* isolates to antifungal drugs has already been described (40–42, 55, 56). Thus, this study aimed to verify the susceptibility and resistance of these organisms to commercial compounds. We also aimed to correlate these results with responses to different propolis extracts. Our results showed that the response of *M. pachydermatis* to commercial antifungals was variable. Most of the propolis extracts were able to inhibit the growth of *M. pachydermatis* isolates and kill these organisms in *in vitro* assays.

The MIC and MFC values for all antifungal drugs were higher than the previously observed values for *M. pachydermatis*. The MIC values of amphotericin B determined in this study differed from those observed in previous reports (52, 53, 56), in which there were no reports of *M. pachydermatis* resistance to this polyene. Values below the suggested cut-off value for itraconazole were reported (41, 42, 50, 52, 55, 57), which were in agreement with 69.23% (9/13) of the isolates tested. The MIC values of ketoconazole were concordant among the reviewed studies, and had low activity variation (41, 42, 55–57). Higher ketoconazole MIC values were observed in only one study (MIC_90_ = 0.5 μg/mL) (52). Lower inhibition was reported using the Etest methodology (ketoconazole MIC values ranging from 1.6–5.2 μg/mL) (44), and was in agreement with our findings. A variable response to fluconazole has already been reported for *Malassezia* clinical isolates (55), and only one of the isolates tested herein had an MIC of 8 μg/mL. The MICs of all other isolates were greater than this value. In contrast to the findings of this study, the MIC values for fluconazole were reportedly lower for *M. pachydermatis* isolated from dogs with (56) and without otitis (43, 55).

A high frequency of azole-resistant isolates was observed in the present study, compared to other findings with strains isolated from dogs with *M. pachydermatis* dermatitis or otitis. Of these isolates, 12.5, 4, and 4.4% were resistant to fluconazole and ketoconazole (9), itraconazole (58), and fluconazole (59), respectively. In another study, a smaller proportion of isolates obtained from dogs and cats were resistant (60). Of these, 2.4 and 3.7% of isolates were resistant to fluconazole and ketoconazole, respectively. An extreme case of resistance was reported for yeast isolated from a human patient with fungemia (fluconazole MIC > 256 μg/mL) (40).

Azoles have been frequently used in veterinary medicine for the treatment of fungal infections, and are the most commonly used antifungals. The prolonged and repetitive treatment required for recurrence-selected resistant isolates accelerates the development of fluconazole resistance (39, 48). The high resistance rate of the clinical isolates may be attributable to this phenomenon of artificial selection. This is because the animals from which the fungi were isolated were housed in a veterinary hospital frequented by a low-income population. In the majority of cases, such individuals are not able to fully treat their animals. These animals are often taken to veterinarians after a long period of infection, commonly during the chronic phase of malasseziosis. Moreover, besides an alternative to common antifungal drugs and preventing resistance development, alternative methods for treating malasseziosis are more affordable for these individuals.

It has been reported that 86.6% of *M. pachydermatis* isolates acquired fluconazole resistance during the experimental period, and isolates developed concomitant resistance to itraconazole (42). The ability to acquire resistance to fluconazole *in vitro*, along with cross-resistance to other azoles such as ketoconazole and itraconazole, has also been reported (39). These findings corroborate the hypothesis of cross-resistance between the azoles observed in the present study. This reinforces the importance of performing susceptibility tests to define the most effective therapy, not only in the clinical practice routine, but also for assay standardization for this specific yeast on an international level.

Despite the importance of *M. pachydermatis* as an etiologic agent against dermatomycoses (45, 46, 60, 61), studies exploring the therapeutic potential of Brazilian propolis extracts against this yeast are scarce (45, 46). The fungicidal activity of a propolis ethanolic extract obtained from Rio Grande do Sul (Brazil) on clinical isolates from dogs with otitis has been reported (MIC_50_ = 2.6 mg/mL and MFC_90_ = 5.3 mg/mL) (45). These results are in agreement with the inhibition showed by the red propolis ethanolic and supercritical extracts. Lower MIC values for these extracts have already been reported (46), and the fungistatic and fungicidal activities were observed in the range of 0.8–2.4 mg/mL. These studies do not mention the color, botanical source, or chemical profile of the propolis used in the tests, and do not allow improved analysis and correlation between their results and the results of our study. Thus, to our knowledge, this is the first study using characterized propolis extracts for the determination of the *M. pachydermatis* resistance/susceptibility profile.

The inhibitory potential of propolis extracts has been more commonly studied for *Candida* species, particularly *C. albicans*, and the MIC results were lower than those observed in our study. Fluconazole-resistant clinical isolates of *C. albicans* were inhibited by an Iranian propolis extract (18). Similar MIC values were reported for Southeast Brazilian type 3 and Northeast type 13 propolis ethanolic extracts and their fractions against *Candida* specimens (17). The red propolis ethanolic extract obtained from Northeast Brazil, especially the acetanolic fraction (MIC ≤ 50 μg/mL), was also effective against *C. albicans* (23). The propolis ethanolic extract and related microparticles obtained from the Paraná State reportedly exhibited fungicidal activity against the multiresistant and dose-dependent response to azoles and amphotericin B *Candida* clinical isolates (62). All propolis extracts inhibited the growth of isolates presenting a dose-dependent response pattern and antifungal resistance, in concordance with our results. The fungicidal and fungistatic activities of Brazilian green and red propolis extracts are also reportedly observed for other fungi genera, such as *Saccharomyces* (63) and *Trichophyton* (64–67).

The inhibition-related results for each propolis extract can be correlated with total phenolic content, total flavonoid content, and antioxidant activity. The phenolic and flavonoid compounds of propolis are considered important for their anti-inflammatory, antimicrobial, and antifungal activities (31, 62). The brown propolis ethanolic extract, which had a lower total phenolic compound and flavonoid content, showed less inhibitory activity compared to that of the red and green propolis extracts. This correlation was also observed for the antibacterial activity of green, red, and brown propolis ethanolic extracts obtained from Bahia, Brazil (31). The antifungal properties of Brazilian propolis type 3 and 13 are attributed to their flavonoid content (17). The green and red propolis analyzed in this study also contained important flavonoids and exhibited greater fungicidal effects.

Flavonoids, especially pinocembrin, are considered to be responsible for inhibitory activity toward the genera *Candida* (68, 69) and *Trichophyton* (66). Formononetin and pinocembrin are the most abundant flavonoid components of red propolis obtained from Northeast Brazil, and our results suggest that formononetin is at least partially responsible for its antimicrobial activity (21). The results of analyzing ethanolic extract and the hexane fraction of type 13 red propolis revealed that formononetin and medicarpine act as chemical biomarkers (17). This is in accordance with the results of previous studies (16, 70). Quercetin and medicarpine are the main compounds responsible for the high antifungal activity observed for propolis type 3 and 13 (17). It is possible that these compounds enhanced the antifungal activity of red and green propolis in our study. The lowest EC_50_ value was observed for supercritical red propolis extract, indicating that the compounds of interest present in this extract may induce a lower level of dose dependence. It was suggested that the mechanism of action of type 13 propolis against yeast was related to cell wall rupture and not to the structure of ergosterol or alterations in plasma membrane permeability (71). This explains the observed sensitivity of isolates with variable resistance profiles to azoles and of polyene derivatives to propolis extracts.

Aside from the fact that red and green propolis extracts exhibited similar inhibitory activities, green propolis ethanolic extract demonstrated fungicidal activity at a lower dose (4 mg/mL). This may be attributable to the fact that Brazilian green propolis contains a significant amount of artepillin C, which has been known to exhibit marked antibacterial and antioxidant activity (10, 72). Artepillin C acts synergistically with phenolic and flavonoid compounds against *Staphylococcus aureus* isolates (72).

One of the major issues arising from the MIC and MFC values obtained herein is whether these concentrations of propolis extract can induce cytotoxicity. It was observed that green and red propolis concentrations of 5–80 μg/mL did not affect *in vitro* macrophage viability. However, cytotoxic effects were observed at green and red propolis concentrations of 160 μg/mL (31). Mohammadzadeh et al. (73) reported that mice that were orally administered with hydroalcoholic extracts of Iranian propolis with concentrations of 4,500–20,000 mg/Kg did not have any clinical or behavioral toxicological changes. Our recent studies have shown that a green propolis-based ointment used for treating surgical wounds in sheep and containing 20% (w/w) of green propolis extract did not induce any toxic effects (74).

In summary, responses to antifungals varied widely among all *M. pachydermatis* isolates, save for amphotericin B. Furthermore, a high rate of resistance was observed for all tested drugs. All propolis extracts inhibited *M. pachydermatis* clinical isolate growth, including those that showed resistance to antifungal agents. Red and green propolis extracts showed the highest fungicidal activity. These results encourage the need for further studies evaluating the inhibitory activity of isolated compounds, in addition to the elucidation of their mechanisms of action via *in vivo* experiments.

## Data Availability Statement

All datasets generated for this study are included in the article/Supplementary Material.

## Author Contributions

KD, MF, CF, and RP carried out the experimental procedures, data preparation and interpretation, and wrote the manuscript. DO carried out the experimental procedures and reviewed the manuscript. LS conducted fungal isolation and culture maintenance and preservation. SH and RM prepared and interpreted the data and wrote the manuscript. BM and MU-G were responsible for obtaining and extracting propolis. RM and RP contributed to manuscript preparation and funding acquisition. All authors read and approved the final manuscript.

### Conflict of Interest

The authors declare that the research was conducted in the absence of any commercial or financial relationships that could be construed as a potential conflict of interest.
