# Supplementary material for: Susceptibility of Malassezia pachydermatis Clinical Isolates to Allopathic Antifungals and Brazilian Red, Green, and Brown Propolis Extracts
Source: Front Vet Sci. 2019 Dec 13;6:460. doi: 10.3389/fvets.2019.00460 (PMC6923270; doi:10.3389/fvets.2019.00460)
Supplement: Supplementary file 1 [file Data_Sheet_1.PDF]

Supplementary material 1 - Determination of humidity content, total solids, total ashes, crude protein, total lipids, crude fibers and water activity (aw) of red, green and brown propolis samples collected in different regions of Brazil. RAL: red propolis from Alagoas, Brazil; GPR: green propolis from Paraná, Brazil; BSC: brown propolis from Santa Catarina, Brazil.

**Sample**	**Humidity (%)**	**Total Solids (%)**	**Total Ashes (%)**	**Protein (%)**	**Lipids (%)**	**Aw (%)**	**Fibers (%)**
RAL	7.03±0.42	92.97±0.42	0.96±0.03	2.30±0.05	66.33±0.01	0.689±0.01	7.66±0.90
GPR	7.13±0.12	92.87±0.12	3.15±0.03	9.98±0.83	48.72±1.29	0.688±0.02	20.89±1.39
BSC	7.07±0.10	92.93±0.10	1.73±0.19	3.90±0.49	74.31±5.69	0.657±0.02	7.29±0.30
