# Supplementary material for: Susceptibility of Malassezia pachydermatis Clinical Isolates to Allopathic Antifungals and Brazilian Red, Green, and Brown Propolis Extracts
Source: Front Vet Sci. 2019 Dec 13;6:460. doi: 10.3389/fvets.2019.00460 (PMC6923270; doi:10.3389/fvets.2019.00460)
Supplement: Supplementary file 2 [file Data_Sheet_2.PDF]

Supplementary material 2. Characterization of the ethanolic and supercritical extracts used in this experiment, as previously described.[10] RAL – SC: red propolis supercritical extract, from Alagoas, Brazil; RAL – ET: red propolis ethanolic extract, from Alagoas, Brazil; GPR – ET: green propolis ethanolic extract, from Paraná, Brazil; BSC – ET: brown propolis ethanolic extract from Santa Catarina, Brazil.

Sample	Extraction method	Conditions	Geographical origin	Type	Total phenolic compounds (mg EAG/g)	Flavonoids (mg EQ/g)	DPPH (IC50)	ABTS (%) (Trolox 1 mg.ml^{-1})
**RAL – SC**	Supercritical	40 °C, 300 bar	Alagoas	Red	157.16±0.01	40.65±0.01	183.11±0.31	82.80±3.50
**RAL – ET**	Conventional	70 °C, ambient pressure	Alagoas	Red	198.77±0.01	58.19±0.01	44.29±0.29	98.20±1.30
**GPR – ET**	Conventional	70 °C, ambient pressure	Paraná	Green	179.52±0.01	39.90±0.01	157.39±0.26	89.90±1.80
**BSC – ET**	Conventional	70 °C, ambient pressure	Santa Catarina	Brown	117.03±0.01	27.97±0.01	163.00±0.31l	89.80±1.20
