# Supplementary material for: Susceptibility of Malassezia pachydermatis Clinical Isolates to Allopathic Antifungals and Brazilian Red, Green, and Brown Propolis Extracts
Source: Front Vet Sci. 2019 Dec 13;6:460. doi: 10.3389/fvets.2019.00460 (PMC6923270; doi:10.3389/fvets.2019.00460)

Supplementary material 8. Growth inhibition (%) of *M. pachydermatis* organisms isolated from the (**A**) skin of dogs with dermatitis and (**B**) ears of dogs and *Didelphis* with otitis was determined using different concentrations of the green propolis ethanolic extract (mg/mL) at the broth microdilution technique. The results for the strain herein used as the reference (BH3) strain are included in both graphs. The results represent the means obtained in two independent experiments.

A

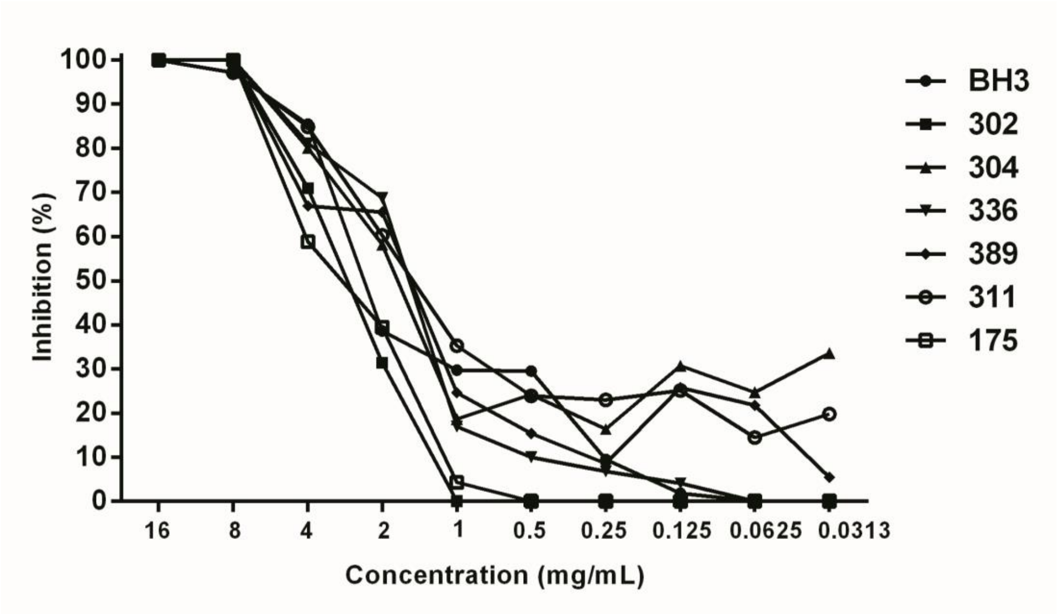

B

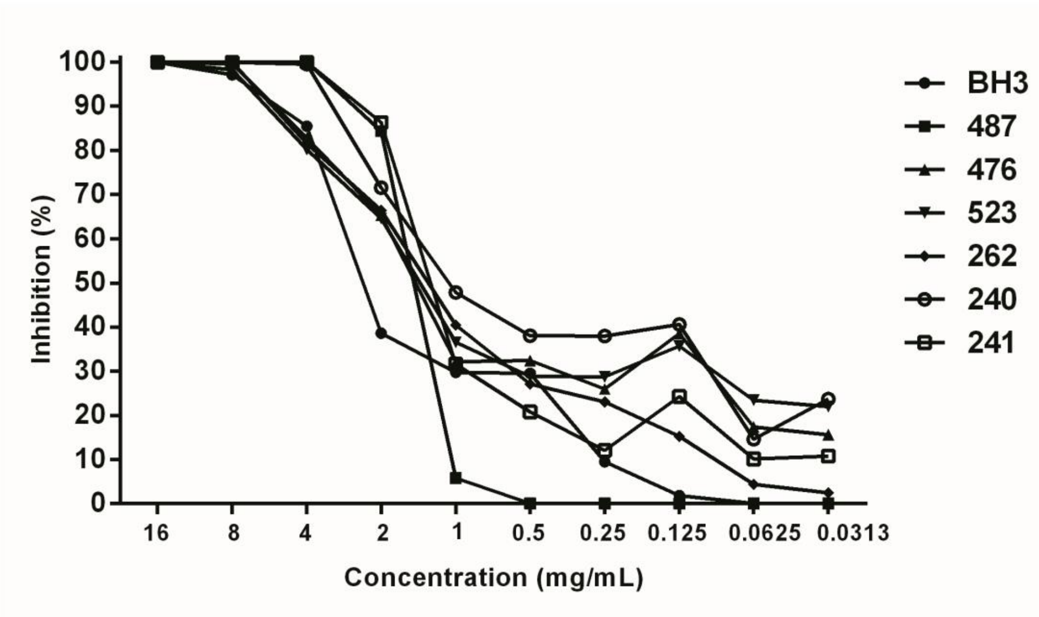

Supplement: Supplementary file 8 [file Data_Sheet_8.PDF]
