# Supplementary material for: Susceptibility of Malassezia pachydermatis Clinical Isolates to Allopathic Antifungals and Brazilian Red, Green, and Brown Propolis Extracts
Source: Front Vet Sci. 2019 Dec 13;6:460. doi: 10.3389/fvets.2019.00460 (PMC6923270; doi:10.3389/fvets.2019.00460)

Supplementary material 10. Growth inhibition (in %) of *M. pachydermatis* organisms isolated from the (**A**) skin of dogs with dermatitis and (**B**) ears of dogs and *Didelphis* with otitis was determined using different concentrations of the red propolis supercritical extract (mg/mL) at the broth microdilution technique. The results represent the means obtained in two independent experiments.

A

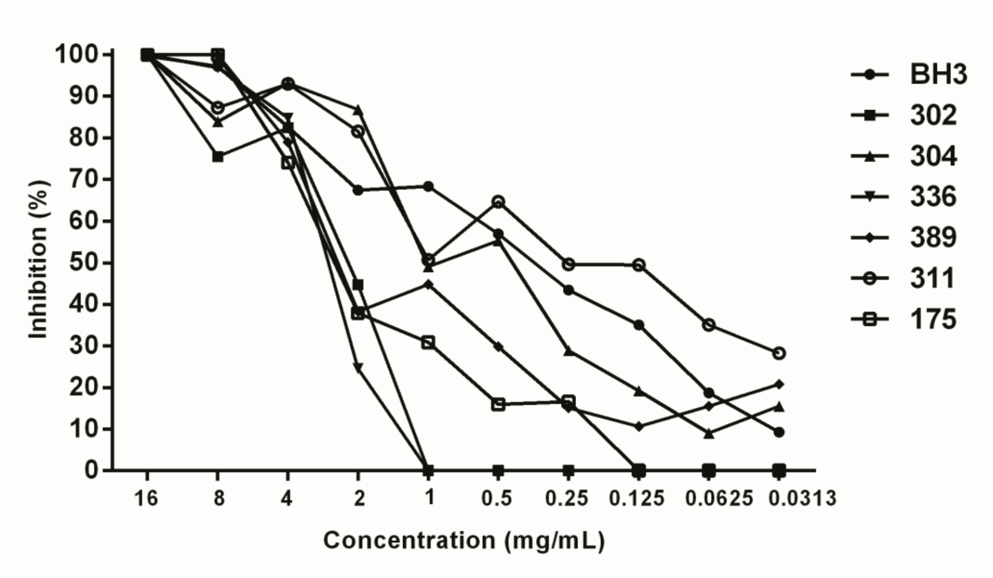

B

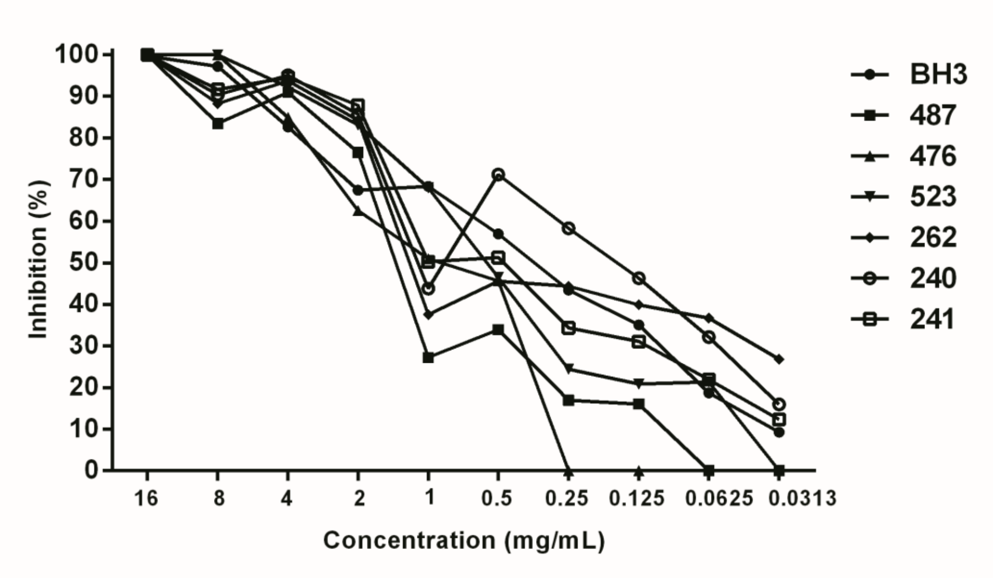

Supplement: Supplementary file 10 [file Data_Sheet_10.PDF]
